# e-Health in Cardiovascular Medicine

**DOI:** 10.3390/medsci7060072

**Published:** 2019-06-20

**Authors:** Julie Redfern, Lis Neubeck

**Affiliations:** 1 University of Sydney, Westmead Applied Research Centre, Faculty of Medicine and Health, Sydney 2006, Australia; 2 The George Institute for Global Health Australia, Sydney 2145, Australia; 3 School of Health and Social Care, Edinburgh Napier University, Edinburgh EH14 1DJ, UK; L.Neubeck@napier.ac.uk

Cardiovascular disease (CVD), including coronary artery disease (CHD) and stroke, is the leading cause of death and disease burden globally [1]. An estimated 17.9 million people died from CVD in 2016 representing 31% of all global deaths [1]. Importantly, 85% of the 2016 deaths due to CVD were due to heart attack and stroke, and three-quarters of all CVD deaths occurred in low- and middle-income countries [1]. However, much of the CVD burden can be prevented by addressing behavioural risk factors such as, tobacco use, unhealthy diet and obesity, physical inactivity and harmful use of alcohol using population-wide strategies [1]. With an increasing world-wide population, more people are living longer, and more people are surviving the initial CVD events, thus increasing the overall CVD burden [2]. Therefore, implementation of prevention and management strategies are a current international health priority [3] as they offer an evidence-based solution for reducing morbidity, mortality and costs [4].

Overall, the increasing global burden of disease is alarming and we are challenged with the need for scalable solutions that address prevention and ongoing management. Solutions lie in changes in the individual, family, communities and broader society. Such solutions need to overcome the barriers of distance, capacity and resourcing, while being simple and meaningful to people. Major advances in affordable technology offer an opportunity to maximise effectiveness and reach of health care. In the past decade, there has been rapid proliferation in the number of people who are able to access the Internet, with 4.1 billion people using the Internet globally, increasing from 3.7 billion Internet users in late 2017 [5]. The escalating use and availability of technology has the potential to expand communication in transformative ways. There are many potential definitions of e-Health, but Eysenbach defines it as: “an emerging field in the intersection of medical informatics, public health and business, referring to health services and information delivered or enhanced through the Internet and related technologies” [6].

As such, e-Health encompasses the provision of health-related services, hardware devices and software. Examples include the use of the Internet, Bluetooth devices, remote monitoring devices, wearable devices, text-messages and smartphone apps, amongst others. In recent years, there has been rapid expansion of these in the development and delivery of strategies aiming to address a number of health needs across different medical conditions. These strategies include education of patients and healthcare providers, data collection, diagnostics and screening, patient monitoring, treatment support, behavioural change support and communication between patients, health professionals and health services [7]. Current and future research is therefore needed to explore development of new and improved devices and strategies, to develop an evidence-base for those that exist and to optimise implementation and translation into routine practice.

The aim of this Special Edition of *Medical Sciences* is to explore potential opportunities for e-Health in CVD research and management. The special edition includes a variety of original research papers and reviews that cover development and implementation of several e-Health strategies. All are aimed at using technology to increase reach and enable better delivery of CVD prevention and management.

The paper by Wang et al. [8] assessed the relationship of hs-troponins with echocardiographic markers of left ventricular hypertrophy and structural heart disease in 78 patients. The study highlights the importance of technology and ongoing research in identification of screening tools that can potentially guide clinical decision-making. The paper by Mondal et al. [9] provides an example of how an e-Health hardware device is initially developed. The work itself outlines the development of a low-cost wireless phonocardiograph with a Bluetooth headset [9]. Importantly, the work emphasises the potential value of e-Health in low resource settings. The developed device is capable of recording sounds while connected to a mobile device with audio recording application, and therefore serves as a wireless phonocardiograph. The system then enables electronic sharing of audio files for remote identification of abnormal heart sounds.

The paper by Dowie et al. [10] reports an approach that utilises e-Health to support evidence-based decision-making for aortic stenosis. In this strategy, the software electronically collates evidence to inform medical decision-making and as such provides an example of how the software can be used to synthesise complex information to support delivery of evidence-based care. The paper by Santo et al. [11] explores patient decision-making in relation to use of apps. The paper presents a mixed-methods qualitative research project, where the patients’ perspectives and views associated with the use of medication reminder apps are explored. The study was conducted alongside the MedApp-CHD randomised controlled trial [12]. The current paper explores the patients’ perspectives in terms of using the apps for targeting medication adherence and what factors influenced their engagement with them.

The paper by Clark et al. [13] highlights how electronic systems and devices can be used to collect data that inform research and health services. The authors present the tracker study that explores the activity levels and utilisation of healthcare services of patients after discharge from hospital for acute coronary syndrome [13]. The authors highlight and discuss the challenges associated with managing the large volume of data collected and provide an example of how real-time data can be used to help understand patient activity. Finally, the paper by Raeside et al. [14] outlines the use of e-Health strategies for primary prevention of CVD in adolescents. This is an important target population who are known to engage in extensive use of mobile technology, and hence understanding and considering how e-Health strategies in this population is of great interest. The concept of e-Health and primary prevention along with the increasing need to focus on co-creation are also interesting concepts explored in the paper.

In conclusion, the current special edition of *Medical Sciences* offers a variety of examples on how e-Health data and strategies can potentially be used to improve the delivery and reach of healthcare. Future research and health services reviews will no doubt continue to explore, define and determine the evidence-base for e-Health and its ultimate contribution in the years to come.
